# Effect of Entecavir Combined with Adefovir Dipivoxil on Clinical Efficacy and TNF-*α* and IL-6 Levels in Patients with Hepatitis B Cirrhosis

**DOI:** 10.1155/2021/9162346

**Published:** 2021-08-24

**Authors:** Yonghuan Yu, Xinfeng Cui, Jingjing Zhao, Ting Jia, Baofeng Ren, Xiaoyan Zhang

**Affiliations:** ^1^Department of Blood Transfusion, Yantaishan Hospital, Yantai 264000, China; ^2^Department of Stomatology, Chengyang People's Hospital, Chengyang 266109, China; ^3^Department of Surgery, Zhangqiu District People's Hospital, Jinan 250200, China; ^4^Department of Gynaecology, Zhangqiu District People's Hospital, Jinan 250200, China; ^5^Medical Insurance Department, Zhangqiu District People's Hospital, Jinan 250200, China; ^6^PIVAS, Weifang People's Hospital, Weifang 261041, China

## Abstract

**Objective:**

The purpose of the study was to investigate the effect of entecavir combined with adefovir dipivoxil on clinical efficacy and TNF-*α* and IL-6 levels in patients with hepatitis B cirrhosis.

**Methods:**

A total of 100 patients with hepatitis B cirrhosis admitted to our hospital between January 2018 and June 2019 were randomly selected and divided into the control group (*n* = 50) and experimental group (*n* = 50) according to the order of admission. Among them, the control group patients were treated with entecavir, while the patients in the experimental group received entecavir combined with adefovir dipivoxil. After that, the effective rate of treatment, the incidence of adverse reactions, liver function indexes, liver fibrosis condition, and TNF-*α* and IL-6 expression levels were all compared between the two groups.

**Results:**

The effective rate of treatment in the experimental group was significantly higher than that in the control group, with statistical significance (*p* < 0.001); the incidence of adverse reactions of the patients in the experimental group was significantly lower than that in the control group, with statistical significance (*p* < 0.001); the liver function indexes in the experimental group were significantly better than those in the control group, with statistical significance (*p* < 0.001); the number of patients with liver fibrosis in the experimental group was significantly less than that in the control group, with statistical significance (*p* < 0.001); the TNF-*α* and IL-6 expression levels in the experimental group were significantly lower than those in the control group, with statistical significance (*p* < 0.001).

**Conclusion:**

Entecavir combined with adefovir dipivoxil in the treatment of hepatitis B cirrhosis can effectively improve the therapeutic effect and reduce the serum inflammatory factor levels, with high safety, which is worthy of application and popularization.

## 1. Introduction

Hepatitis B cirrhosis refers to clinically common chronic liver cirrhosis caused by hepatitis B, which poses a great threat to people's daily life [1]. Due to the infection of hepatitis B virus (HBV), the liver function of patients is affected to some extent, resulting in a higher risk of liver cirrhosis [2–4]. Liver fibrosis refers to the pathological process of abnormal hyperplasia of connective tissue in the liver, which is an important factor affecting the prognosis of chronic liver disease. Clinical trials have found that patients with hepatitis B cirrhosis may show liver fibrosis and increased expression levels of serum inflammatory factors during treatment, leaving them in a microinflammatory state for a long time and thus affecting the therapeutic effect [5]. Entecavir, adefovir dipivoxil, and lamivudine, with antibacterial and antivirus effects, are common and effective clinical drugs to treat liver cirrhosis [6]. Although entecavir has a good antiviral effect for newly diagnosed patients with hepatitis, they are easily affected by high variability of the virus. In addition, long-term administration will increase drug resistance and reduce the therapeutic effect. Therefore, the combination of entecavir with other drugs in the treatment of hepatitis B cirrhosis has become a hot spot in clinical research [7]. Adefovir dipivoxil is a pentacyclic purine nucleotide analogue that inhibits HBV replication [8]. In order to explore the better treatment method for patients with hepatitis B cirrhosis, patients with hepatitis B cirrhosis were selected as the research objects of this study, and different treatment methods were adopted to compare the effective rate of treatment, the incidence of adverse reactions, liver fibrosis condition, and the expression levels of inflammatory factors, specifically reported as follows.

## 2. Materials and Methods

### 2.1. General Information

A total of 100 patients with hepatitis B cirrhosis admitted to our hospital between January 2018 and June 2019 were randomly selected and divided into the control group (*n* = 50) and experimental group (*n* = 50) according to the order of admission, with aging from 26 to 66 years old in the experimental group and from 25 to 67 years old in the control group. There was no statistical significance in the comparison of general information such as gender and age, between the two groups (*p* > 0.05), as given in Table 1.

### 2.2. Inclusion/Exclusion Criteria

#### 2.2.1. Inclusion Criteria

- Patients met the diagnostic criteria of hepatitis B cirrhosis in basic and clinical features of hepatitis B cirrhosis [9] and were confirmed by pathological or imaging diagnosis
- Patients were 18 years of age or older
- Patients had no history of drug allergy, drug abuse, and bad addiction
- Patients had no other organic diseases
- This study was approved by the hospital ethics committee, and the patients all voluntarily participated in this study and signed the informed consent.

#### 2.2.2. Exclusion Criteria

- Patients had disturbance of consciousness and could not cooperate with the study
- Patients suffered from liver cancer
- Patients with severe hepatic and renal dysfunction

### 2.3. Methods

All patients underwent routine physical examinations. 3 ml of fasting venous blood examples was collected from the patients to evaluate their liver and kidney functions and routine blood indexes.

All patients orally took entecavir tablets (Manufacturer: Sino-American Shanghai Squibb Pharmaceutical Co., Ltd.; NMPA approval no. H20052237; specification: 0.5 mg), 1 tablet each time, once a day, for 24 weeks.

The patients in the experimental group were additionally treated with adefovir dipivoxil (Manufacturer: Fujian Cosunter Pharmaceutical Co., Ltd.; NMPA approval no. H20070198; specification: 10 mg) orally, 1 tablet each time, once a day, for 24 weeks.

### 2.4. Observation Indexes

The effective rate of treatment, the incidence of adverse reactions, liver function indexes, liver fibrosis condition and TNF-*α* and IL-6 expression levels were all compared between the two groups.

#### 2.4.1. Serum Detection

5 ml of fasting venous blood was collected from patients in both groups after treatment, and the upper serum was taken after centrifugation. The serum TNF-*α* and IL-6 expression levels were determined by the enzyme-linked immunosorbent assay (ELISA). The kits were purchased from Kamai Shu (Shanghai) Biotechnology Co., Ltd., and the operation was strictly conducted according to the kit instructions. The normal range was 740–1540 pg/ml for TNF-*α* and 56.37–150.33 pg/ml for IL-6.

The markedly effective referred to that patients' clinical manifestations and the detection of hepatitis B virus basically disappeared and liver function obviously improved; the effective referred to that patients' clinical manifestations obviously relieved, the detection of hepatitis B virus obviously decreased, and liver function improved; the ineffective referred to that patients' clinical manifestations had no obvious remission but aggravation, the detection of hepatitis B virus had no obvious decrease, and liver function had no improvement. Total effective rate = (the number of markedly effective + the number of effective)/total number × 100%.

Liver function test indexes included serum total protein (TP), glutamic-pyruvic transaminase (ALT), glutamic-oxaloacetic transaminase (AST), total bilirubin (TBIL), and total bile acid (TBA) [10–12].

#### 2.4.2. Diagnosis of Liver Fibrosis

After treatment, all patients received ultrasound-guided liver biopsy, and the number of cases with liver fibrosis was compared between the two both groups.

### 2.5. Statistical Treatment

The selected data processing software for this study was SPSS20.0 (IBM, Armonk, NY, USA), and GraphPad Prism 7 (GraphPad Software, San Diego, USA) was used to draw the pictures of the data. Measurement data were expressed by ($\bar{x}\pm s$) and tested by the *t*-test. Enumeration data were expressed as (*n* (%)) and tested by the *X*^2^ test. The differences had statistical significance when *p* < 0.05.

## 3. Results

### 3.1. Comparison of the Effective Rate of Treatment between the Two Groups

The comparison of the effective rate of treatment between the two groups showed that the effective rate of treatment of 94% in the experimental group was significantly higher than that of 76% in the control group, with statistical significance (*p* < 0.05), as given in Table 2.

### 3.2. Comparison of the Incidence of Adverse Reactions between the Two Groups

The patients suffered from ascites, gastrointestinal bleeding, and portal hypertension during treatment, and the incidence of adverse reactions in the experimental group was significantly lower than that in the control group, with statistical significance (*p* < 0.05), as given in Table 3.

### 3.3. Comparison of Liver Function between the Two Groups

The comparison of liver function between the two groups showed that liver function test indexes in the experimental group were all significantly better than those in the control group, with statistical significance (*p* < 0.05), as given in Table 4.

### 3.4. Comparison of Liver Fibrosis Condition between the Two Groups

**Figure 1:**
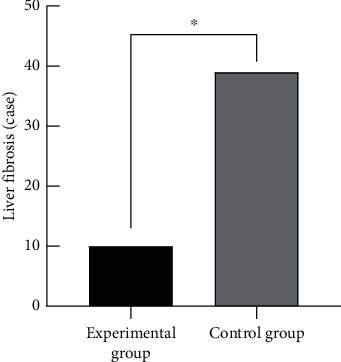
Comparison of liver fibrosis condition between the two groups (*n* (%)). Note: the abscissa represented the experimental group and control group, while the ordinate represented the cases of liver fibrosis. ^*∗*^The comparison between 10 patients with liver fibrosis in the experimental group and 39 patients with liver fibrosis in the control group, *X*^2^ = 33.65, *p* < 0.001, with statistical significance.

The number of patients suffering from liver fibrosis in the two groups was recorded and compared, and the results showed that the number of patients suffering from liver fibrosis in the experimental group was significantly lower than that in the control group, with statistical significance (*p* < 0.05), as shown in Figure 1.

### 3.5. Comparison of TNF-*α* and IL-6 Expression Levels between the Two Groups

**Figure 2:**
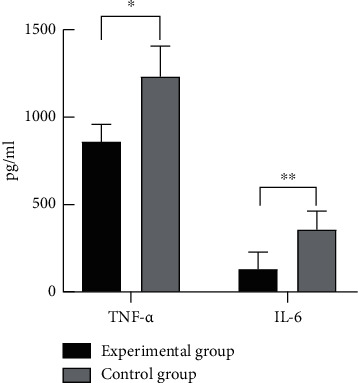
Comparison of TNF-*α* and IL-6 expression levels between the two groups ($\bar{x}\pm s$). Note: the abscissa represented TNF-*α* and IL-6, while the ordinate represented the expression level (pg/ml). ^*∗*^The comparison of TNF-*α* expression levels between the experimental group of (862.38 ± 100.01) pg/ml and the control group of (1237.25 ± 174.16) pg/ml, *t* = 13.20, *p* < 0.001, with statistical significance. ^*∗∗*^The comparison of IL-6 expression levels between the experimental group of (129.66 ± 98.24) pg/ml and the control group of (357.19 ± 105.39) pg/ml, *t* = 11.17, *p* < 0.001, with statistical significance.

The comparison of TNF-*α* and IL-6 expression levels between the two groups revealed that the TNF-*α* and IL-6 expression levels in the experimental group were significantly lower than those in the control group, with statistical significance (*p* < 0.05), as shown in Figure 2.

## 4. Discussion

Further spread of hepatitis B virus (HBV) in human body can cause liver dysfunction and cirrhosis, and the majority of the patients with hepatitis B will be affected by cirrhosis and liver dysfunction, posing a great threat to their physical health [13–15]; therefore, timely treatment should be carried out to prevent the development of related complications. With antibacterial, anti-infection, and anti-HBV functions, drugs such as entecavir and adefovir dipivoxil are commonly used for hepatitis B cirrhosis, and their main components were guanine nucleoside analogues, which can inhibit hepatitis B polymerase and have been confirmed in the treatment of chronic hepatitis B [16]. Liver fibrosis is a pathological repairing reaction of the liver to chronic injury. The further development of liver fibrosis can cause the disorder of liver structure, nodular regeneration of liver cells, and formation of cirrhosis. Therefore, it is of great significance to effectively control the process of liver fibrosis in patients and improve the prognosis of patients [17, 18]. Although both drugs have good therapeutic efficacy in clinical treatment, their effect on controlling liver fibrosis and inflammatory factors in patients is not obvious [15, 19]. In order to deeply investigate the effective treatment method and analyze the clinical efficacy of the combination of entecavir and adefovir dipivoxil, in this study, the patients with hepatitis B cirrhosis were selected as the study subjects to explore the effects of entecavir combined with adefovir dipivoxil as well as entecavir dipivoxil alone on liver function, inflammatory factor levels, and so on.

Our study results showed that the effective rate of treatment in the experimental group was significantly higher than that in the control group, with statistical significance (*p* < 0.05), indicating that entecavir combined with adefovir dipivoxil can greatly improve the therapeutic effect. In this study, the evaluation indexes of the effective rate of treatment included the liver function test, clinical manifestations, and hepatitis B virus testing, and the results revealed that entecavir combined with adefovir dipivoxil could significantly relieve patients' clinical manifestations, eliminate hepatitis B virus, and improve their liver function and that the liver function indexes in the experimental group were significantly better than those in the control group, with statistical significance (*p* < 0.05). TNF-*α* and IL-6 are proinflammatory factors with the synergistic effect, which can promote the progress of liver fibrosis and inflammation in patients, while the incidence of adverse reactions in patients is the key index to evaluate the effect of drug therapy [20]. In addition, the cases of liver fibrosis, the incidence of adverse reactions, and serum TNF-*α* and IL-6 expression levels in the experimental group were significantly lower than those in the control group (*p* < 0.05), which was similar to the results of Ren et al. [21] who have pointed in their study that entecavir combined with adefovir dipivoxil has significantly better efficacy in reducing HBeAg-negative chronic hepatitis B than entecavir alone. All these demonstrate that entecavir combined with adefovir dipivoxil can significantly reduce the incidence of adverse reactions, inflammatory factors expression levels, and the incidence of liver fibrosis during the treatment of hepatitis B cirrhosis. Regina et al. [22] in their studies have pointed out that entecavir combined with adefovir dipivoxil can obviously increase the effective rate in the treatment of hepatitis B cirrhosis combined with liver fibrosis and can effectively improve liver fibrosis and liver function; therefore, entecavir combined with adefovir dipivoxil in patients with hepatitis B cirrhosis combined with liver fibrosis has a high application value, which is consistent with the conclusion of this study, fully demonstrating the scientific reliability of the findings of this study. This study also has some limitations. For example, the sample size is small, the influence of disease staging on the results is not considered, and long-term efficacy is not included in this study.

In conclusion, entecavir combined with adefovir dipivoxil in the treatment of hepatitis B cirrhosis can effectively improve the therapeutic effect and reduce the serum inflammatory factor levels, with high safety, which is worthy of popularization.

## Figures and Tables

**Table 1:** Comparison of general information between the two groups (*n* (%), $\bar{x}\pm s$).

Group	Experimental group	Control group	*X*^2^/*t*	*P*
Gender (male/female)	23/27	26/24	0.36	0.55
Age (years old)	43.39 ± 6.21	43.77 ± 6.39	0.30	0.76
Height (cm)	165.32 ± 10.01	165.64 ± 10.37	0.16	0.88
Weight (kg)	71.45 ± 5.90	71.39 ± 5.64	0.05	0.96
Disease course (months)	5.68 ± 1.69	5.72 ± 1.73	0.12	0.91
Smoking history (years)	4.31 ± 1.33	4.27 ± 1.38	0.15	0.88
Drinking history (years)	10.96 ± 1.38	10.52 ± 1.22	1.69	0.09
Hypertension (cases)	13	15	0.20	0.66
Diabetes (cases)	8	7	0.08	0.78
Hyperlipidemia (cases)	4	6	0.44	0.51

**Table 2:** Comparison of the effective rate of treatment between the two groups (*n* (%)).

Group	Markedly effective	Effective	Ineffective	Total effective rate (%)
Experimental group	33	14	3	94%
Control group	10	28	12	76%
*X* ^2^	—	—	—	6.35
*P*	—	—	—	0.01

**Table 3:** Comparison of the incidence of adverse reactions between the two groups (*n* (%)).

Group	Ascites	Gastrointestinal bleeding	Portal hypertension	Total incidence (%)
Experimental group	1	1	0	4%
Control group	3	5	8	32%
*X* ^2^	—	—	—	13.28
*P*	—	—	—	<0.001

**Table 4:** Comparison of liver function between the two groups ($\bar{x}\pm s$).

Group	TP (g/L)	ALT (U/L)	AST (U/L)	TBIL (*μ*mol/L)	TBA (*μ*mol/L)
Experimental group	73.59 ± 3.66	24.39 ± 2.74	30.66 ± 3.48	15.52 ± 2.51	5.21 ± 0.52
Control group	66.75 ± 2.51	97.71 ± 5.68	77.61 ± 4.45	23.69 ± 3.30	11.48 ± 2.23
*t*	10.90	82.21	58.77	13.93	19.36
*P*	<0.001	<0.001	<0.001	<0.001	<0.001

## Data Availability

The data used to support the findings of this study are available from the corresponding author upon request.

## References

[1] Pol S., Lusivika N. C., Carrat F. (2021). Letter: tenofovir may be superior to entecavir for treatment‐naïve chronic hepatitis B patients—authors’ reply. *Alimentary Pharmacology & Therapeutics*.

[2] Liu Y., Chen R., Liu W. (2021). Investigation of multidrug-resistance mutations of hepatitis B virus (HBV) in a large cohort of chronic HBV-infected patients with treatment of nucleoside/nucleotide analogs. *Antiviral Research*.

[3] Mak L.-Y., Huang Q., Wong D. K.-H. (2021). Residual HBV DNA and pgRNA viraemia is associated with hepatocellular carcinoma in chronic hepatitis B patients on antiviral therapy. *Journal of Gastroenterology*.

[4] Naz A., Tabish I., Naseer A., Siddiqi A. Z., Siddiqui F. A., Mirza A. Z. (2021). Green chemistry approach: method development and validation for identification and quantification of entecavir using FT-IR in bulk and pharmaceutical dosage form. *Future Journal of Pharmaceutical Sciences*.

[5] Hui V. W.-K., Yip T. C.-F., Wong V. W.-S. (2021). Aspirin reduces the incidence of hepatocellular carcinoma in patients with chronic hepatitis B receiving oral nucleos(t)ide analog. *Clinical and Translational Gastroenterology*.

[6] Kakiuchi T., Takahashi H., Iwane S., Koji A., Matsuo M. (2021). Entecavir administration to pregnant Japanese woman with chronic hepatitis B and hepatocellular carcinoma: a case report. *Clinical case reports*.

[7] Yang S., Ma X., Cai C., Wang H., Xiao F., Yu C. (2021). Tenofovir disoproxil fumarate is superior to entecavir in reducing hepatitis B surface antigen for chronic hepatitis B in China: 2-year comprehensive comparative result of a matched comparative study. *Frontiers in Medicine*.

[8] Papatheodoridis G. V., Dalekos G. N., Idilman R. (2020). Similar risk of hepatocellular carcinoma during long-term entecavir or tenofovir therapy in Caucasian patients with chronic hepatitis B. *Journal of Hepatology*.

[9] Lin C.-L., Tseng K.-C., Chen K.-Y., Liao L.-Y., Kao J.-H. (2020). Factors predicting outcomes of hepatitis B-related cirrhosis patients with long-term antiviral therapy. *Journal of the Formosan Medical Association*.

[10] Ma J.-L., He L.-L., Li P., Jiang L., Wei H.-S. (2020). “Prognosis of endotherapy versus splenectomy and devascularization for variceal bleeding in patients with hepatitis B-related cirrhosis. *Surgical Endoscopy*.

[11] Ni L., Li C., Li Y. (2020). Correlation of APOBEC3G expression with liver function indexes of patients with chronic hepatitis B and comparison in chronic hepatitis B, liver cirrhosis and liver cancer. *Oncology letters*.

[12] Su T.-H., Peng C.-Y., Tseng T.-C. (2020). Serum mac-2-binding protein glycosylation isomer at virological remission predicts hepatocellular carcinoma and death in chronic hepatitis B-related cirrhosis. *The Journal of Infectious Diseases*.

[13] Jin X., Li X. (2020). Factors predicting outcomes of hepatitis B-related cirrhosis patients with long-term antiviral therapy. *Journal of the Formosan Medical Association*.

[14] Yang J. Q., Zeng R., Cao J. M. (2019). Predicting gastro-oesophageal variceal bleeding in hepatitis B-related cirrhosis by CT radiomics signature. *Clinical Radiology*.

[15] He L., Ye X., Ma J. (2019). Antiviral therapy reduces rebleeding rate in patients with hepatitis B-related cirrhosis with acute variceal bleeding after endotherapy. *BMC Gastroenterology*.

[16] Hung T.-H., Tsai C.-C., Lee H.-F. (2019). Population-based study of entecavir and long-term mortality in chronic hepatitis B–related decompensated liver cirrhosis. *Clinics and Research in Hepatology and Gastroenterology*.

[17] Zhang L. L., Li Y. F., Zhang C., Wu X. F., Ma Y., Li L. (2019). Study on the risk of hepatitis B-related cirrhosis combined with type 2 diabetes mellitus for the occurrence of primary hepatocellular carcinoma. *Zhonghua Gan Zang Bing Za Zhi = Zhonghua Ganzangbing Zazhi = Chinese Journal of Hepatology*.

[18] Wang J., Zhang Z., Yan X. (2019). Albumin-Bilirubin (ALBI) as an accurate and simple prognostic score for chronic hepatitis B-related liver cirrhosis. *Digestive and Liver Disease*.

[19] Ren M., Li J., Xue R., Wang Z., Li Coll S., Meng Q. (2019). Liver function and energy metabolism in hepatocellular carcinoma developed in patients with hepatitis B-related cirrhosis. *Medicine*.

[20] Lian M. J., Zhang J. Q., Chen S. D., Zhang D. D., Yang Y. Y., Hong G. L. (2019). Diagnostic accuracy of *γ*-glutamyl transpeptidase-to-platelet ratio for predicting hepatitis B-related fibrosis: a meta-analysis. *European Journal of Gastroenterology & Hepatology*.

[21] Ren M., Li J., Xue R., Wang Z., Coll S. L., Meng Q. (2019). Liver function and energy metabolism in hepatocellular carcinoma developed in patients with hepatitis B-related cirrhosis. *Medicine*.

[22] Zhao C., Niu H., Regina (202). “Effects of entecavir combined with adefovir dipivoxil on liver fibrosis and serum ALT and FFA levels in patients with chronic hepatitis B. *Systemic Medicine*.

